# A Double Herd Krill Based Algorithm for Location Area Optimization in Mobile Wireless Cellular Network

**DOI:** 10.1155/2015/475806

**Published:** 2015-03-23

**Authors:** F. Vincylloyd, B. Anand

**Affiliations:** ^1^RVS Technical Campus, Coimbatore 641 402, India; ^2^Hindusthan College of Engineering and Technology, Coimbatore 641 032, India

## Abstract

In wireless communication systems, mobility tracking deals with determining a mobile subscriber (MS) covering the area serviced by the wireless network. Tracking a mobile subscriber is governed by the two fundamental components called location updating (LU) and paging. This paper presents a novel hybrid method using a krill herd algorithm designed to optimize the location area (LA) within available spectrum such that total network cost, comprising location update (LU) cost and cost for paging, is minimized without compromise. Based on various mobility patterns of users and network architecture, the design of the LR area is formulated as a combinatorial optimization problem. Numerical results indicate that the proposed model provides a more accurate update boundary in real environment than that derived from a hexagonal cell configuration with a random walk movement pattern. The proposed model allows the network to maintain a better balance between the processing incurred due to location update and the radio bandwidth utilized for paging between call arrivals.

## 1. Introduction

Location areas represent a significant strategy of location management, used to reduce signalling traffic imposed by location updating and paging messages in mobile cellular networks. Due to the increasing dimension spaces to be searched, the location of optimal LAs represents a NP-hard optimization problem. In contrast to a landline telephonic network, mobile wireless cellular network (MWCN) accommodates dynamically relocatable service users with whom location uncertainty is always associated. To reduce this location uncertainty, each mobile terminal has to report its location information in regular interval, which is called an LU procedure. In dynamic LU scheme, the frequency of LU performed by a mobile terminal (MT) depends upon a stochastic phenomenon, which is user's movement behavior [1–6].

Upon the arrival of a mobile-terminated call, it is the responsibility of the network to search for the terminal for delivering the call successfully. This search is an iterative process, which continues until the terminal is successfully located. The frequency of paging to be performed by the network, per user, depends upon another stochastic phenomenon, which is incoming call arrival process for each user [7].

Since LU and paging process both consume sufficient amount of radio resource, cost is incurred for performing an LU as well as for paging. Both of these processes are coupled in a sense that there is an inherent trade-off between these two cost components, and these two together determine the total network cost. The size of the LA, in particular, affects the signalling load generated due to paging and LU. From a designer's point of view, it is required to find out an optimum size of LA such that the desired cost effectiveness can be achieved.

More precisely, the location area (LA) planning represents a vital role in cellular networks because of the trade-off created by paging and registration signaling. The upper bound on the size of an LA is the service area of a mobile switching center (MSC). In that extreme case, the cost of paging is at its highest, but no registration is needed. On the other hand, if each cell is an LA, the paging cost is minimal, but the registration cost is the most. In general, the most important component of these costs is the load on the signaling resources. Between the extremes lie one or more partitions of the MSC service area that minimize the total cost of paging and registration. The present work falls into the class of location area planning (LAP) problem [8, 9].

For hexagonal cell configuration, [10] had tried to find out optimum movement threshold value, which would, in effect, determine the number of cells, which collectively could be considered as a dynamic optimal LA (as it depends upon two stochastic phenomena, namely, call arrival pattern and microscopic behavioural pattern of terminal mobility). Due to the uniqueness of design considerations and problem formulation in [10], they used GA and SA method to solve the problem and have compared the quality of solutions found by each for different inputs.

In fact, there is a growing body of literature in the application of emerging heuristics to solve the optimizing problems in various fields of science and engineering, but there is a huge vacuum in the application of heuristics for the location area (LA) problem. This paper proposes a Krill Herd based algorithm for minimizing total network cost, comprising location update (LU) cost and cost for paging. Krill herd algorithm (KHA) is a recently developed powerful evolutionary algorithm proposed by Gandomi and Alavi [11]. The KHA is based on the herding behaviour of krill individuals. Each krill individual modifies its position using three processes, namely, (1) movement induced by other individuals, (2) foraging motion, and (3) random physical diffusion.

Although some may disagree that a suitable algorithm design would assure a high probability of finding solution, population size does indirectly contribute to the effectiveness and efficiency of the performance of an algorithm [12]. The prime deciding factor of population size on any population-based heuristic algorithms is the execution cost. If an algorithm involves large population size, it will search thoroughly and increase the chance of exploring the entire search space and locating possible good solutions but unavoidably bear an unwanted and high computational cost. The other version is if an algorithm with small population size may suffer from premature convergence or may search partially the search space. Perhaps suggesting heuristically a suitable population size may be adequate because one need not know the exact fitness landscape to solve a complex optimization problem. Hence, a compromised, yet effective, solution would be dynamically adjusting the population size to explore the search space in balance between computational cost and the attained performance [13].

In this paper, the basic KHA is enhanced by incorporating a dual population criterion to find an optimal solution of the above problem. There are few literatures that tackle the issue of population size with various heuristics [14–16]. The rest of paper is organized as follows. In Section 2, the system as well as the proposed model in [10] is revisited. In Section 2.3, the constrained cost optimization problem for LA planning is mathematically formulated. In Section 3.1, the KHA technique is overviewed in general and then the KHA based algorithm is proposed for solving the problem of interest. In Section 3.1.1, the dual herd KHA based algorithm is discussed. In Section 4.2, some representative results are presented.  Section 5 concludes the present work.

## 2. System and Model Description

### 2.1. System Description

**Figure 1:**
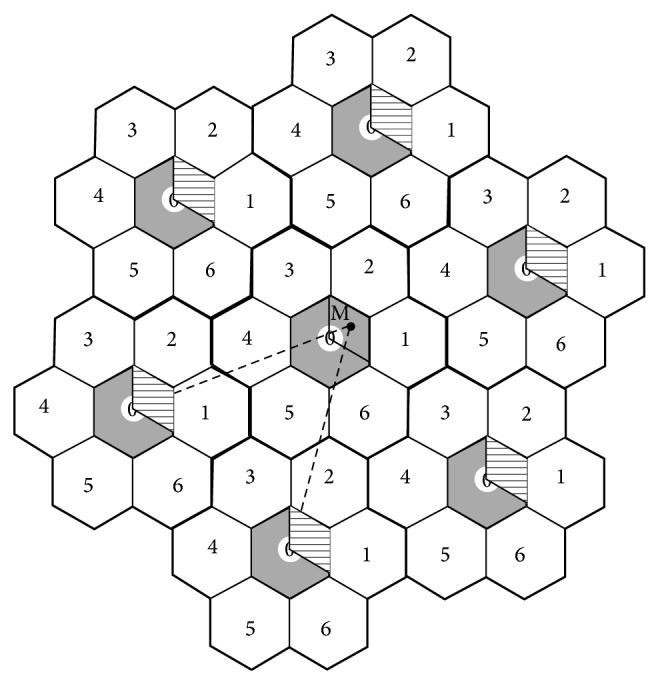
Cellular network architecture for the movement of MTs.

Figure 1 shows that the cellular network coverage area is comprised of hexagonal shaped cells. The entire coverage area is partitioned into rings of cells. The center cell is defined to be a cell where an MT has performed the last LU. An MT resides in each cell it enters, for a generally distributed time interval and then it can move to any of the neighbouring cells. The movement of an MT is assumed to be a simple random walk [17]. The next LU is performed by the MT, when the number of cell boundary crossings, since the last LU, equals a threshold value *d*. It is also assumed that MTs move in a radial direction as shown in Figure 1.

If an MT makes, say, *d* movements in one particular radial direction, as shown in Figure 1, then the LA will be defined as the area within (*d* − 1) rings from the center cell. If *d* assumes an optimum value, then the corresponding LA will be an optimum LA [18].

If the LA consists of *D* rings, then

(1) $$\begin{array}{c} D=\left(d-1\right). \end{array}$$

The number of cells within the LA, *L*_*D*_ will be

(2) $$\begin{array}{c} N\left(D\right)=3*\left(D+1\right)*D+1. \end{array}$$

The perimeter *L*(*D*) of the LA *L*_*D*_, can be calculated as

(3) $$\begin{array}{c} L\left(D\right)=\left(12D+6\right)*R, \end{array}$$

where *R* denotes the radius of the circle inscribing a hexagonal cell and the area of each cell is $(3\sqrt{3}/2)R^{2}$ and

(4) $$\begin{array}{c} d=2\sqrt{3}R. \end{array}$$

The area *S*(*D*) of the LA *L*_*D*_ is

(5) $$\begin{array}{c} S\left(D\right)=\left[3D*\left(D+1\right)+1\right]*\left(2.6\right)*R. \end{array}$$

Next, it is assumed that the incoming call arrivals to each MT follow a Poisson process. The paging area is the entire LA that is the area within (*d* − 1) rings from the center cell.

### 2.2. Analytical Model

Let *α*(*k*) be the probability that there are *K* boundary crossings performed by an MT between two successive call arrivals. If the probability density function of cell residence time *t*_*m*_ has the Laplace-Stieltjes transform *F*_m_^*^(*s*) MT and mean 1/*λ*_*m*_, the call arrival to each terminal follows Poisson process with rate *λ*_*c*_. Based on these assumptions, the expression of *α*(*k*) can be derived as follows [6]. Here, *θ* is the call-to-mobility ratio (CMR), where CMR is defined as *λ*_*c*_/*λ*_*m*_ [6]:

(6) $$\begin{array}{c} \alpha \left(k\right)=\left\{\begin{array}{cc} 1-\frac{1}{\theta }\left[1-F_{m}^{*}\left(\lambda_{c}\right)\right], & K=0,\\ \frac{1}{\theta }{\left[1-F_{m}^{*}\left(\lambda_{c}\right)\right]}^{2}{\left[F_{m}^{*}\left(\lambda_{c}\right)\right]}^{K-1}, & K>0. \end{array}\right. \end{array}$$

Considering that cell residence time *t*_*m*_ follows a Gamma distribution, we get [6]

(7) $$\begin{array}{c} F_{m}^{*}\left(s\right)={\left[\frac{\left(\lambda_{m}\gamma \right)}{\left(s+\lambda_{m}\gamma \right)}\right]}^{\gamma }, \end{array}$$

where

(8) $$\begin{array}{c} \gamma =\frac{1}{\left(v_{\text{ar}}\lambda_{m}^{2}\right)}. \end{array}$$

Let *β*(*k*, *K*) denote the probability that the MT is *k* rings away from the center cell, given that the mobile user has already performed *K* number of cell boundary crossings. To depict the mobility pattern of an MT, a 2D random walk model is considered. Let us assume that *P*_*K*_ denotes the *K* × *K* state transition matrix, where an element *p*_*i*,*j*,*k*_ in *P*_*K*_ gives us the probability that a mobile terminal moves from one *i*th ring cell to one *j*th in single step. Then, the probability *β*(*k*, *K*) comes out to be [6]

(9) $$\begin{array}{c} \beta \left(k,K\right)=\left\{\begin{array}{cc} P_{K}; & \text{Single step state transition matrix,}\\ P_{K}^{*}P_{K}^{\left(n-1\right)}; & n\;\;\text{step transition matrix.} \end{array}\right. \end{array}$$

Since we want to find out the optimum value of *d*, we express LU cost *C*_*u*_ as a function of *d* and the expression is as follows (detailed derivation is given in [19]):

(10) $$\begin{array}{c} C_{u}\left(d\right)=\frac{U^{*}\left(Y\left[1-F_{m}^{*}\left(\lambda_{C}\right)\right]/\theta \right)*\left({\left[F_{m}^{*}\left(\lambda_{c}\right)\right]}^{d-1}\right)}{{\left(1-{\left[F_{m}^{*}\left(\lambda_{C}\right)\right]}^{d}\right)}^{2}}. \end{array}$$

Similarly, we derive paging cost *C*_*v*_ as a function of *d* and it is as follows (detailed derivation is in [19]):

(11) $$\begin{array}{c} C_{v}\left(d\right)=V*\overset{d-1}{\underset{k=0}{\sum }}\rho_{-k}N\left(k\right)\pi \phi_{k}, \end{array}$$

where *Y* is the number of MT attached to the network within the LA and *U* and *V* are respective cost coefficients for performing an LU and paging and *ϕ*_*k*_ is the probability of finding the MT within the LA, *L*_*k*_; the density of the MT in that area is denoted by *ρ*_*k*_. So the total cost is

(12) $$\begin{array}{c} C_{T}\left(d\right)=C_{u}\left(d\right)+C_{v}\left(d\right) \end{array}$$

or

(13) CTd=U∗Y1−Fm∗λC/θ∗Fm∗λCd−11−Fm∗λCd2 +V∑k=0d−1ρ−kNkϕk.

### 2.3. Problem Formulation

The constrained optimization problem can be stated mathematically as

(14) Minimize   P:CTd=Cud+Cvd

(15) Subject to:  0<p<1

(16)       qkSkρk=Nkp

(17)       R<Rmax⁡.

In constraint (15), *p* denotes the penetration factor. Constraint (16) gives the total number of MTs in a service area where *ρ*_*k*_ is the density of the MTs in an LA *L*_*k*_, and there are *q*_*k*_ switches. This number must be greater than or equal to the total number of attached mobile users. In this constraint *N*_*k*_ denotes population size within the LA. The maximum radius is to be considered so that the constraint on system power budget is not violated with respect to (17).

## 3. Solution Methodology

The high complexity associated with the optimization problem (*P*) necessitates computationally efficient and robust tools. Traditional techniques like gradient search, linear programming, quadratic programming, and so forth were not fruitful due to the complexity involved in the problem. This encouraged us to propose two heuristics which are based on krill herd algorithm and are presented in the next section. However, the quality of solution (compared to classical optimization techniques) is often traded off against computation time, and one can always reach a near optimal solution within bounded computation time.

### 3.1. Krill Herd Algorithm: An Overview

Krill herd algorithm (KHA) is a recently developed heuristic algorithm based on the herding behavior of krill individuals. It has been first proposed by Gandomi and Alavi [11]. It is a population-based method consisting of a large number of krill in which each krill moves through a multidimensional search space to look for food. In this optimization algorithm, the positions of krill individuals are considered as different design variables and the distance of the food from the krill individual is analogous to the fitness value of the objective function. In KHA, the individual krill alters its position and moves to the better positions. The movement of each individual is influenced by the three processes, namely, (i) induction process, (ii) foraging activity, and (iii) random diffusion. These operators are briefly explained and mathematically expressed as follows.

*(i) Induction.* In this process, the velocity of each krill is influenced by the movement of other krill individuals of the multidimensional search space and its velocity is dynamically adjusted by the local, target, and repulsive vector. The velocity of the *i*th krill at the *m*th movements may be formulated as follows [19]:

(18) vim=αivimax⁡+ωnvim−1,αi=∑j=1NSfi−fjfw−fb×Zi−ZjZi−Zj+rand0,1+2rand0,1+iimax⁡fibestZibest,

where *V*_*i*_^max⁡^ is the maximum induced motion; *V*_*i*_^*m*^ and *V*_*i*_^*m*−1^ are the induced motion of the *i*th krill at the *m*th and (*m* − 1)th movement; *ω*_*n*_ is the inertia weight of the motion induced; *f*_*w*_ and *f*_*b*_ are the worst and the best positions, respectively, among all krill individuals of the population; *f*_*i*_ and *f*_*j*_ are the fitness value of the *i*th and *j*th individuals, respectively; *N*_*S*_ is the number of krill individuals surrounding the particular krill; *i* and *i*_max⁡⁡_ are the current iteration and the maximum iteration number.

A sensing distance (SD_*i*_) parameter is used to identify the neighboring members of each krill individual. If the distance between the two individual krill is less than the sensing distance, that particular krill is considered as neighbor of the other krill. The sensing distance may be represented by [19]

(19) $$\begin{array}{c} {\text{SD}}_{i}=\frac{1}{5n_{p}}\overset{n_{p}}{\underset{k=1}{\sum }}\left|Z_{i}-Z_{k}\right|, \end{array}$$

where *n*_*p*_ is the population size; *Z*_*i*_ and *Z*_*k*_ are the position of the *i*th and *k*th krill, respectively.

*(ii) Foraging Action.* Each individual of krill updates its foraging velocity according to its own current and previous food location. The foraging velocity of the *i*th krill at the *m*th movement may be expressed by [19]

(20) Vfim=0.0221−iimax⁡fi∑k=1NSZk/fk∑k=1NS1/fk+fibestXibest+ωxVfim−1,

where *ω*_*X*_ is the inertia weight of the foraging motion; *V*_*f*_*i*__^*m*−1^ and *V*_*f*_*i*__^*m*^ are the foraging motion of the *i*th krill at the (*m* − 1)th and *m* movement. 

*(iii) Random Diffusion.* In KHA algorithm, in order to enhance the population diversity, random diffusion process is incorporated in krill individuals. This process maintains or increases the diversity of the individuals during the whole optimization process. The diffusion speed of krill individuals may be expressed as follows [19]:

(21) $$\begin{array}{c} v_{D_{i}}^{m}=\mu V_{D}^{\max}, \end{array}$$

where *V*_*D*_^max⁡^ is the maximum diffusion speed; *μ* is a directional vector uniformly distributed between (−1,1). 

*(iv) Position Update.* In KHA, the krill individuals fly around in the multidimensional space and each of krill adjusts its position based on induction motion, foraging motion, and diffusion motion. In this way, KHA combines local search with global search for balancing the exploration and exploitation. The updated position of the *i*th krill may be expressed as [19]

(22) $$\begin{array}{c} Z_{i}^{m+1}=Z_{i}^{m}+\left(V_{i}^{m}+V_{f_{i}}^{m}+V_{D_{i}}^{m}\right)P_{t}\overset{N_{d}}{\underset{j=1}{\sum }}\left(u_{j}-l_{j}\right), \end{array}$$

where *N*_*d*_ is the number of control variables; *u*_*j*_ and *l*_*j*_ are the maximum and minimum limits of the *j*th control variable; *P*_*t*_ is the position constant factor. The above procedure will be used to optimize (14) for location area (LA).

In order to speed up the convergence property and to find better results, the crossover and mutation operations of DE are combined with the proposed algorithm to utilize the exploration ability of DE. These two operators are briefly described below.

#### 3.1.1. Mechanism of the Dual Herd Algorithm

This mechanism is adopted from [15], which uses this for the differential evolution (DE) algorithm. Specifically, this search mechanism is adopted to maintain the population diversity of the krill herd. Also, the original krill herd algorithm is a good local search optimizer [11].

**Step 1 (initialization).**

This step initializes the population for exploration of the search space or else an equal of the herd truncated from the previous initialization will be taken.

**Step 2 .**

Evaluating the new positions using

(23) $$\begin{array}{c} Z_{i}^{m+1}=Z_{r1}^{m}+F*\left(Z_{r2}^{m}-Z_{r3}^{m}\right), \end{array}$$

where *F* = rand(0.01,1) and *r*1, *r*2, and *r*3 are random integers generated from truncated herd.

**Step 3 .**

If rand(0,1) ≥ CR and *j* ≠ *k*, then set *Z*_*i*_^*m*+1^ = *Z*_*i*_^*m*^, where CR = rand(0,1).

**Step 4 .**

Or if rand(0,1) < PM, then *Z*_*i*_^*m*+1^ = rand(LB_*j*_, UB_*j*_), where PM = 0.5.

**Step 5 .**

Repeat from Step 2, until all the individuals in the truncated herd are updated.

The above procedure will ensure the algorithms thorough search of the solution space as it confirms a random choice of candidates [16] compared to a directional policy.

#### 3.1.2. A Double Herd Krill (DHK) Algorithm Developed for Constrained Optimization Problem (*P*)

The proposed double herd KH (DHKH) algorithm problem of interest in this paper is described as follows.

*Pseudocode for the DHK Algorithm*

**Step 1 (data structures).**

Defining the simple bounds, determining algorithm parameter(s), and so forth.

**Step 2 (initialization).**

Randomly creating the initial population in the search space.

**Step 3 (fitness evaluation).**

Evaluation of each krill individual according to its position.

**Step 4 .**

Motion calculation.

**Step 5 .**

Motion induced by the presence of other individuals:

 foraging motion,
 physical diffusion.
 Implement the updating steps of the dual herd algorithm (Section 3.1.1).

**Step 6 (updating).**

Updating the krill individual position in the search space.

**Step 7 (repeating).**

Go to Step 3 until the stop criteria are reached.

**Step 8 .**

End

Krill herd algorithm is applied twice in order to maintain the diversity in the search process. In a single herd algorithm, the search process towards the location area attempts to get hold with that of the local optimal before reaching the convergent solution. As a result, krill herd algorithm is carried out twice which involves diversity in search process and increases the exploration capability during search mechanism resulting in better convergence on the solution in comparison with that of the single krill herd algorithm considered for finding the optimal solution.

## 4. Performance Analysis

In this section, first we have varied some system parameters to observe their reflections on the model behavior, without considering any constraints. Next, we have run our optimization algorithms and have presented some representative results. Lastly, we have compared the performance of the heuristics proposed.

### 4.1. Model Variation

**Figure 2:**
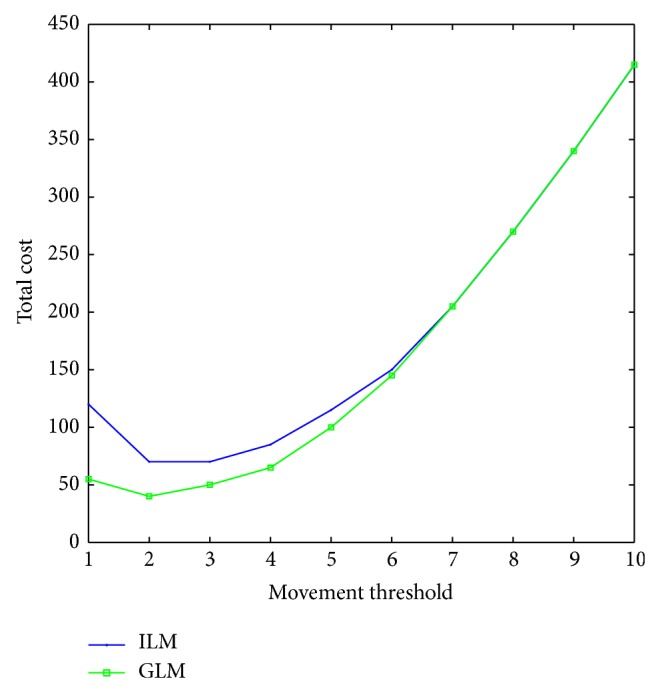
LU cost for various CMR values.

**Figure 3:**
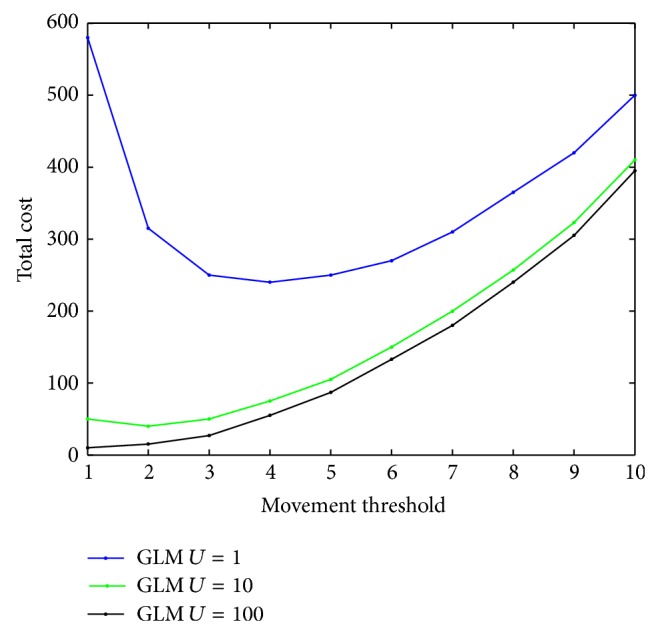
Paging cost for various CMR values.

**Figure 4:**
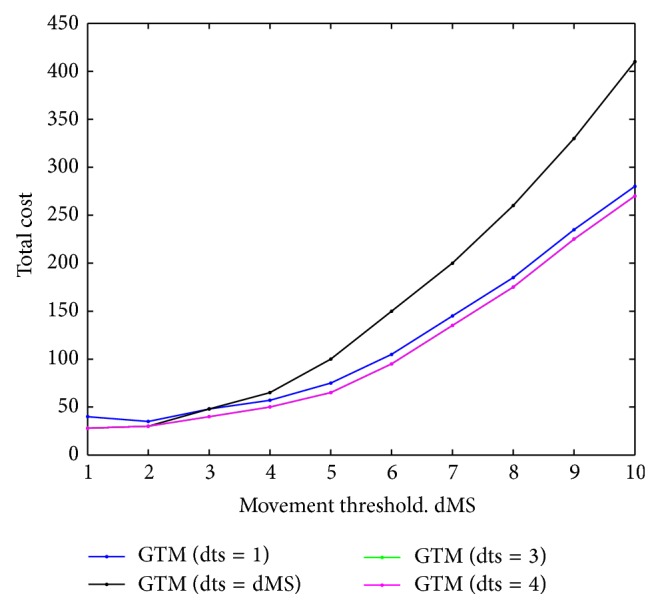
Total cost for various CMR values.

To validate the analytical model, we have varied the value of movement threshold *d* and obtained nature of variation of LU cost, paging cost, and total cost (Figures 2–4), without considering any constraint. The objective of this endeavour is to validate our model as well as our proposed heuristics with respect to that cited in [6]. Through this effort, we find out the region within the entire search space where optimal or near optimal solution could be found.

We take three different values of CMR to demonstrate the effect of changing mobility and call arrival patterns. For a very small value of *d*, the LU cost is very high as lesser number of *d* implies that the frequency of LU is higher and vice versa. This fact can be corroborated from Figure 2. From Figure 2, it has also been seen that, for different CMR values, the LU cost is different. Low CMR means the probability of boundary crossing is high by an MT between two successive calls, which results in higher location update cost. For higher CMR values, the situation is just the reverse. Paging cost also varies as *d* changes. If the value of *d* is small, the size of the LA will be small and the number of cells within the LA will be less. It has been observed in Figure 3 that, for small values of *d*, paging cost is minimal.

As we expected, the paging cost also varies with CMR. If the CMR is high, call arrival rate is high and for each call meant for a mobile terminal, the network will have to go through the call delivery process, which will raise the paging cost significantly.

The total cost *C*_*T*_ varies as *d* changes, which is plotted in Figure 4. For smaller value of *d*, the total cost is high. As we have already explained, even though the paging cost is less, the LU cost is very high. For certain value of *d*, the total cost attains a minimum value, which is the optimum value of *d*. From Figure 4, we find that, for the unconstrained problem for three different values of CMR, the optimum value of *d* lies in the interval [2, 4] when we used our heuristics. The nature of the cost variation with *d* as well as with CMR is the same as presented in [6] with delay equal to one.

### 4.2. Results on Optimization of (*P*)

In this section, we present the results obtained after solving the constrained optimization problem (*P*) using KHA and DHKA. To evaluate the performance of the LAP problem, we have varied the population and network size as well as CMR and have attempted to find out optimum cost for these varying input parameters. We have assumed that in our microcellular configuration each hexagonal cell has a radius of 1 km. Moderate user density (50 users/km^2^) is considered. To calculate the cost coefficient for LU (*U*) is 1 and that for paging (*V*) is 1. The parameter settings for the proposed krill herd algorithm are delineated in Table 1. These values are commonly used for both krill herd algorithms used in this research.

**Figure 5:**
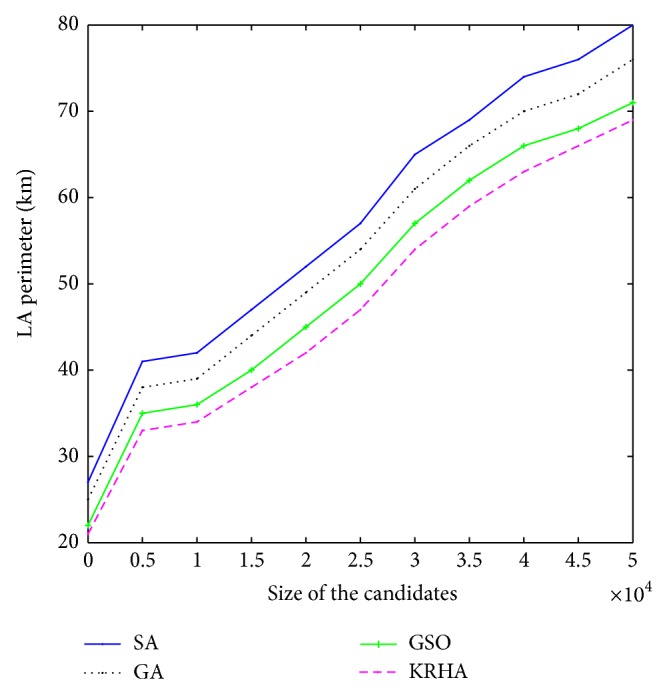
Variation of perimeter of location area for change in candidate size.

Figure 5 shows the variation of perimeter of optimum LA with varying population size. If the population size is increased, optimum value of *d* increases and hence the size of the LA is increased. It is obvious that if larger number of MTs, serviced by a single MSC, are to be accommodated in a single LA, the number of MTs residing closer to the boundary of an LA will be high. Due to this, the possibility of number of boundary crossings by mobile terminals also increases. To minimize the total cost, the movement threshold value *d* would also increase so that after each boundary crossing one LU does not take place. However, Figure 5 shows that DHKA results in slightly smaller LA. For a population size of 800, DHKA finds an optimum *d* value, which is 5.26% less than that generated by SA. However, for a population size of 20,000, using GA, the optimum value that we obtain is 7.9% less than that obtained by running SA.

**Figure 6:**
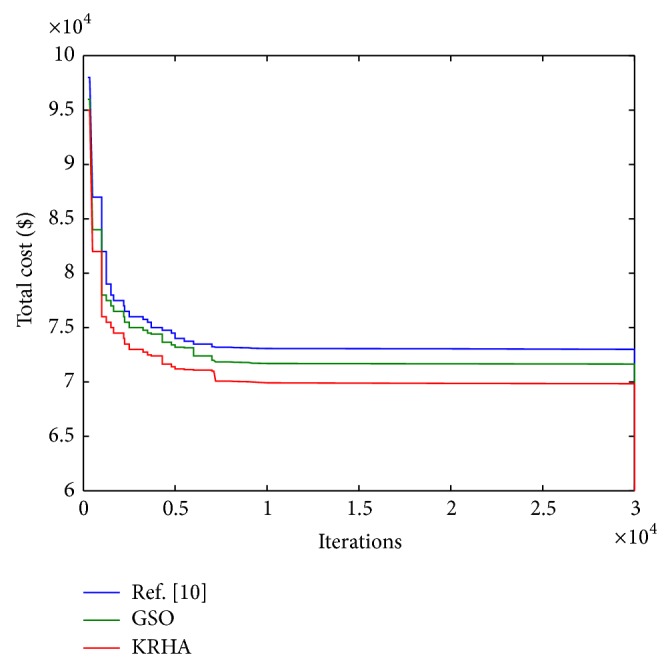
Variation of the optimum total cost for various CMR values.

Figure 6 shows the nature of variation of the optimum LU cost, paging cost, and the total cost with number of cells per LA with *λ*_*C*_ = 0.1 and CMR = 0.1. In this case, as done in [10], the simulation set-up varied the network size consisting of 7 cells to 469 cells corresponding to movement threshold values in the interval [1, 17]. However, while presenting the results, we have rounded up the values of the number of cells per LA to avoid any confusion. For cell size 37 onwards, the LU cost is almost insignificant. However, the paging cost increases as the size of an LA increases.

From Table 2, it is apparent that an LA consisting of cells in the interval [19, 20] results in minimum total cost. Performancewise, both algorithms generate results which are comparable; average paging cost with DHKA came out to be 0.13% less than that obtained by KHA; average total cost only varies by 0.12%. Variation in LU cost is almost negligible. Here, all the LU cost, paging cost, and subsequently the total cost are comparatively less when the cell number is 25. Unlike the results in [10], this research has identified a new set of results, confirming that the location area problem minimization is much effectively solved using the DHKA algorithm compared to the GA and SA methods in [10].

**Figure 7:**
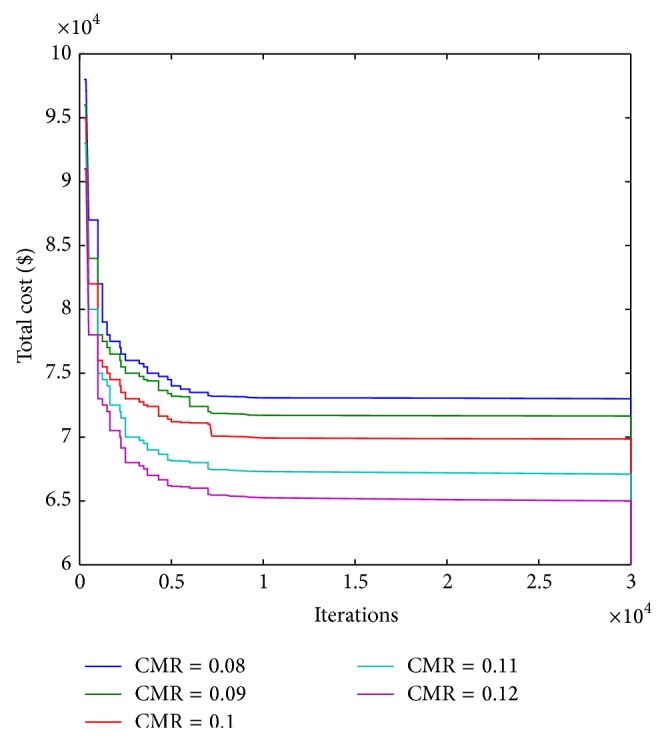
Variation of the optimum total cost using various techniques.

Next, we have varied the value of CMR to investigate its impact on optimum total cost per call arrival. The nature of the variation is shown in Figure 7, where the optimum cost per call arrival decreases with increase in CMR. It is apparent that, for low CMR, call arrival rate is low and the mobility rate of the MT is high. In this situation, the possibility of boundary crossings by an MT increases. This would increase the LU cost significantly, whereas paging cost will be minimal. As CMR increases, the LU cost drastically decreases and so does the total cost. Optimum movement threshold value *d* and hence the perimeter of an LA obtained DHKA come out to be less than those SSA calculates. This pattern becomes apparent as CMR value increases from 0.025 onwards.

### 4.3. Comparative Study of the Performance of the Proposed Solution Methodology

To assess the performance of the two heuristics we have used in this research, we have compared the computation time taken by each algorithm to converge by varying population size and network as well.

**Figure 8:**
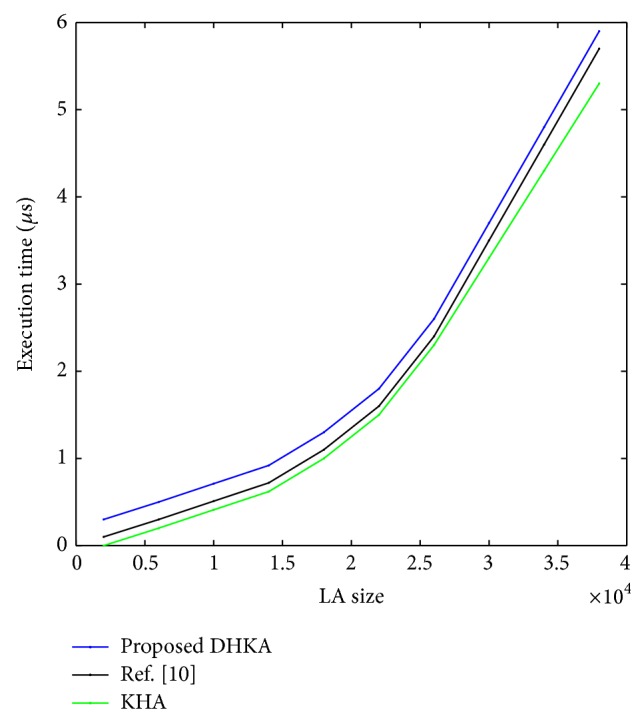
Simulation time of the proposed techniques with respect to LA size (Dummy).

**Figure 9:**
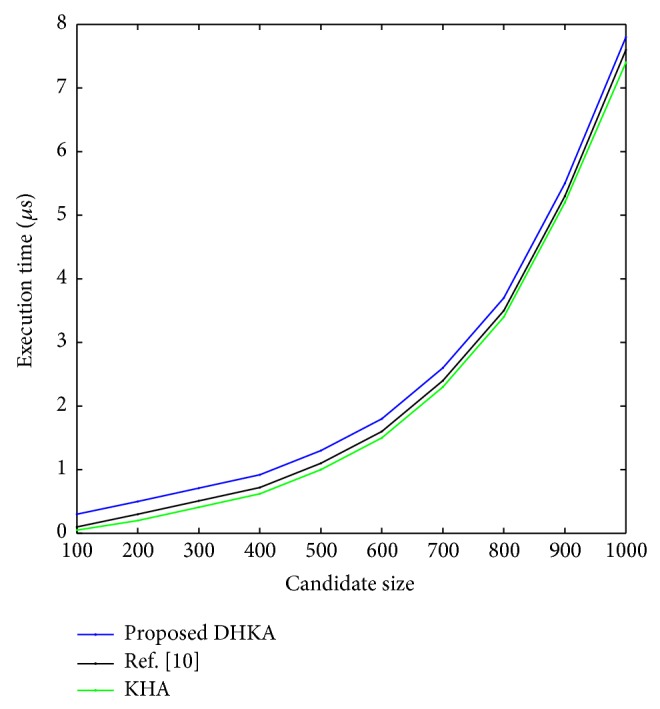
Simulation time of the proposed techniques with respect to candidate size (Dummy).

Figures 8 and 9 show that, even as the network size grows, the proposed KHA based algorithm tends to converge within tolerable run time, whereas DHKA takes much more time to converge. DHKA basically has twice the size of the herd compared to KHA and it continues to search for the best member scanning through generations. The population size and the maximum number of generations conventionally assume large values for DHKA to work properly. On average, KHA based algorithm takes about 0.05 *μ*sec whereas DHKA takes around 4.9 *μ*sec. Despite this fact, DHKA is a strong contender as a combinatorial optimization tool because of its robustness.

## 5. Conclusion

This paper discusses the problem of ever increasing concern for reduction of signalling loads generated in future mobile wireless cellular network (MWCN) due to paging and LU. In this regard, the implication of proper planning for location area (LA) towards optimality is demonstrated. The optimal LA problem was overviewed formally and mathematically formulated considering various practical constraints. Based on various mobility patterns of users and network architecture, the design of the LR area is formulated as a combinatorial optimization problem. The nondifferentiable nature of the problem of interest in this paper has created room for some efficient, robust, and nontraditional search algorithms. This research has proposed a double krill herd algorithm wherein the krill herd is divided based on their global search and local search mechanisms derived from DE. Numerical simulation witnesses promising results, with the condition of widely varying input parameters, some of which are documented. Thus, the proposed DHKA appeared to be a strong alternative for a complex, hard NP-complete optimization problem, such as LAP.

## Figures and Tables

**Table 1:** Parameter settings for the DHK algorithm.

Parameter	Value
Number of krill in the population (KN)	40
Number of iterations (IN)	6000
Foraging speed *V*_*f*_	0.02
Diffusion speed *D*_max⁡_	0.006
Initial violation tolerance (*ε*)	1.0
Decrement (dec)	1.002
Ω_min⁡_	0
*ω*_max⁡_	1

**Table 2:** Results obtained for LA minimization problem using various methods.

No. of cells	Method	LU cost	Paging cost	Total cost
13	[10]	189521	18547	208068
	KHA	188654	18332	206986
	DHKA	188551	18254	206805

19	[10]	87661	19365	107026
	KHA	87421	19122	106543
	DHKA	87225	19085	106310

25	[10]	46442	22541	68983
	KHA	46102	22269	68371
	DHKA	45894	21687	67581

31	[10]	25647	44221	69868
	KHA	25362	44095	69457
	DHKA	25112	43885	68997

37	[10]	17889	53252	71141
	KHA	17567	53054	70621
	DHKA	17339	52985	70324
